# WXG100 Protein Superfamily Consists of Three Subfamilies and Exhibits an α-Helical C-Terminal Conserved Residue Pattern

**DOI:** 10.1371/journal.pone.0089313

**Published:** 2014-02-26

**Authors:** Christian Poulsen, Santosh Panjikar, Simon J. Holton, Matthias Wilmanns, Young-Hwa Song

**Affiliations:** 1 EMBL-Hamburg, Hamburg, Germany; 2 University Luebeck, Institute of Physics, Luebeck, Germany; 3 Australian Synchrotron, Clayton, Victoria, and Department of Biochemistry and Molecular Biology, Monash University, Victoria, Australia; Centre National de la Recherche Scientifique, Aix-Marseille Université, France

## Abstract

Members of the WXG100 protein superfamily form homo- or heterodimeric complexes. The most studied proteins among them are the secreted T-cell antigens CFP-10 (10 kDa culture filtrate protein, EsxB) and ESAT-6 (6 kDa early secreted antigen target, EsxA) from *Mycobacterium tuberculosis.* They are encoded on an operon within a gene cluster, named as ESX-1, that encodes for the Type VII secretion system (T7SS). WXG100 proteins are secreted in a full-length form and it is known that they adopt a four-helix bundle structure. In the current work we discuss the evolutionary relationship between the homo- and heterodimeric WXG100 proteins, the basis of the oligomeric state and the key structural features of the conserved sequence pattern of WXG100 proteins. We performed an iterative bioinformatics analysis of the WXG100 protein superfamily and correlated this with the atomic structures of the representative WXG100 proteins. We find, firstly, that the WXG100 protein superfamily consists of three subfamilies: CFP-10-, ESAT-6- and *sag*EsxA-like proteins (EsxA proteins similar to that of *Streptococcus agalactiae*). Secondly, that the heterodimeric complexes probably evolved from a homodimeric precursor. Thirdly, that the genes of hetero-dimeric WXG100 proteins are always encoded in *bi*-cistronic operons and finally, by combining the sequence alignments with the X-ray data we identify a conserved C-terminal sequence pattern. The side chains of these conserved residues decorate the same side of the C-terminal α-helix and therefore form a distinct surface. Our results lead to a putatively extended T7SS secretion signal which combines two reported T7SS recognition characteristics: Firstly that the T7SS secretion signal is localized at the C-terminus of T7SS substrates and secondly that the conserved residues YxxxD/E are essential for T7SS activity. Furthermore, we propose that the specific α-helical surface formed by the conserved sequence pattern including YxxxD/E motif is a key component of T7SS-substrate recognition.

## Introduction

Bacterial pathogens secrete virulence factors that are involved in processes such as modulation of the host immune system and host cell invasion [1]. Much attention has been focused on the secreted T-cell antigens CFP-10 (10 kDa culture filtrate protein) and ESAT-6 (6 kDa early secreted antigen target) from *Mycobacterium tuberculosis*. The two genes *esxB* encoding for CFP-10 and *esxA* for ESAT-6 are located in the region of difference 1 (RD1), a gene cluster also named as ESX-1 that is essential for the virulence of *M. tuberculosis* [2]. The gene products of ESX-1 assemble a novel bacterial secretory apparatus entitled the type VII secretion system (T7SS) which is responsible for the secretion of CFP-10 and ESAT-6 (Fig. S1) [3], [4]. Interestingly, *M. tuberculosis* possesses five such paralogous loci, ESX-1 to ESX-5, encoding for five T7SSs along with five pairs of *esxB*-like and *esxA*-like genes. Furthermore, *M. tuberculosis* contains six additional *esx*A/B-like tandem genes without accompanied T7SS gene cluster (for nomenclature see [5]). CFP-10 and ESAT-6 are prototypes and the most studied members of the WXG100 protein superfamily. WXG100 proteins are a group of proteins of approximately ∼100 residues in length that contain a conserved “Trp-Xaa-Gly (WXG)” motif [6]. The WXG-motif is centrally positioned within the amino acid sequence and it is almost 100% conserved. Therefore, it can be viewed as a signature for this protein superfamily. Other Gram-positive human pathogens belonging to the phylum Firmicutes, e.g. *Bacillus anthracis* (*B. anthracis*) and *Staphylococcus aureus* (*S. aureus*), also secrete immune potent WXG100 proteins [7]–[9]. For these bacteria, a weakly homologous secretion system designated Type VIIb secretion system (T7SSb) has been proposed (Fig. S1) [5], [10]. Some members of the WXG100 superfamily have already been characterised at the structural level. For example, the NMR structure of the CFP-10/ESAT-6 complex shows a heterodimeric four-helix bundle with flexible termini [11]. Also the atomic structure of the homodimeric WXG100 protein (*sau*EsxA) from *S. aureus* is known [12]. More recently, we solved the X-ray structure of the same complex (CFP-10/ESAT-6). In agreement with the earlier NMR study, it adopts a similar fold of a four-helix bundle [13]. An important question is the general secretion recognition signal of this protein family and its structural features. An initial report on the secretion signal has shown that the seven C-terminal residues of CFP-10 are required for the secretion of the CFP-10/ESAT-6 complex by the T7SS [14]. This report pointed to a secretion signal located at the C-terminus. However, majority of those seven residues are not shared by other WXG100 proteins. More recently, Daleke and co-workers reported a general target signal of the T7SS with the signature motif YxxxD/E, in which the residues Y and the acidic residues (D/E) are crucial for secretion by the T7SS. Moreover, the exact three residues spacing between the Y and D/E is an absolute requirement for substrates of the T7SS [9]. This motif is located at the C-terminus of PE (Pro-Glu motif) proteins (e.g. PE25) and is also shared by other T7SS substrates, such as CFP-10 like proteins and EspB (ESX-1 substrate protein B or Esx secretion associated protein) [9], [15].

In this contribution, we revisited our X-ray structures of the CFP-10/ESAT-6 complex (PDB: 3FAV, [13]). In contrast to the solution structure, the X-ray structure shows better ordered termini which adopt extended α-helical structures. The C-terminal residues of CFP-10 exhibit alpha helical structure up to residue 90 (chain-C in PDB: 3FAV). In addition, we solved the structure of a homologous WXG100 protein from *Streptococcus. agalactiae* (*S. agalactiae*), which forms a homodimer and has similar structural features (PDB: 3GWK, this work and 3O9O [16]). The latter structure resembles that of the homodimeric WXG100 protein from *S. aureus* [12]. Despite the overall structural conservation, the sequence identity among the WXG100 proteins is only about 15% [6]. Therefore, a major focus of this work was to understand the phylogeny of WXG100 proteins and to decode the basis of homodimer and heterodimer formation. Here, we show a complete sequence alignment together with a phylogenetic analysis of WXG100 proteins. In addition, we correlate these observations with the results of our structural analyses of WXG100 proteins showing that the reported general targeting signal YxxxD/E (by Daleke et al. [9]) forms a part of the C-terminal α-helix.

## Results

### Collection of the Non-redundant Set of WXG100 Proteins Using an Iterative Bioinformatics Approach

**Figure 1:**
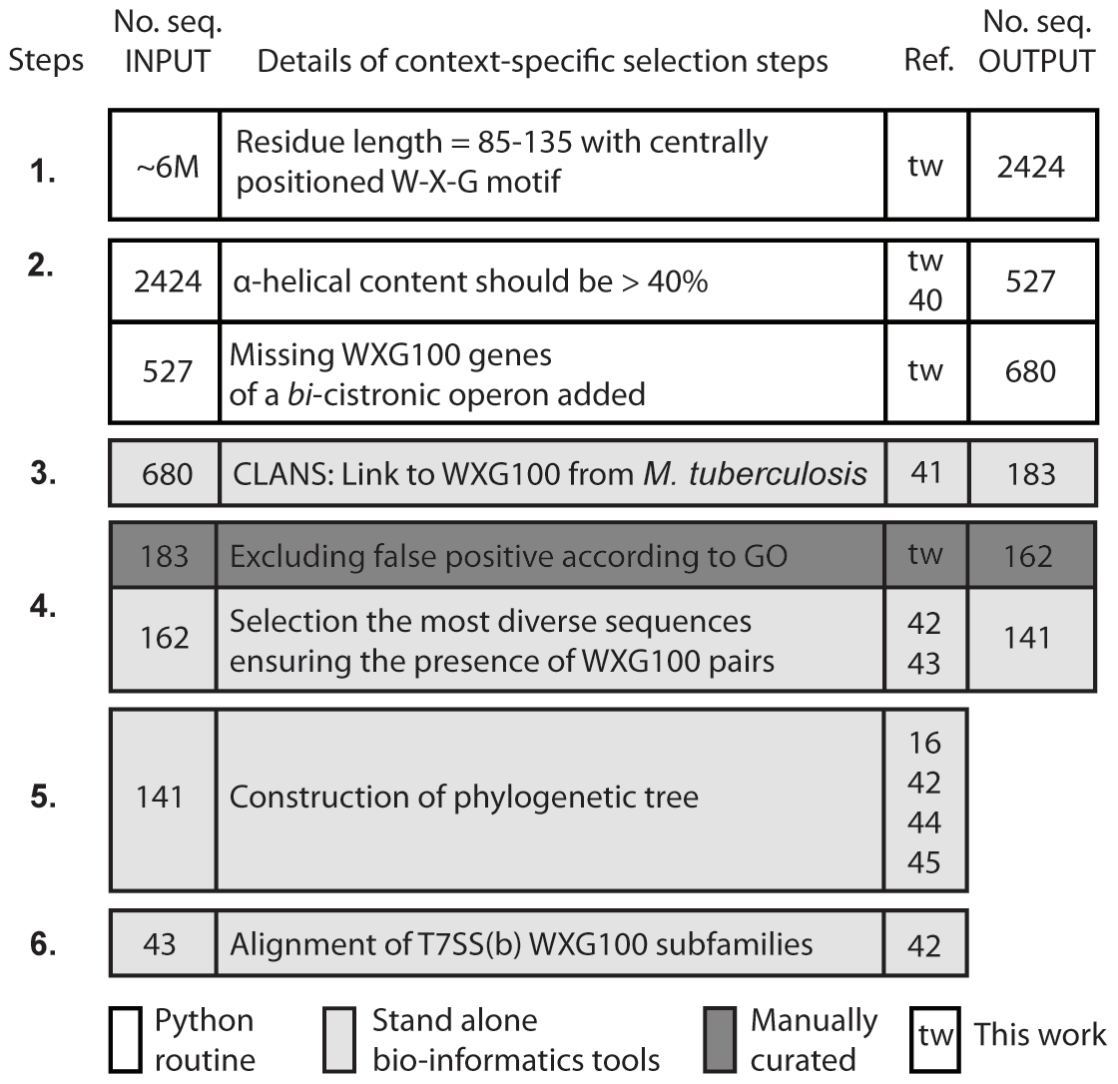
Steps involved in the ‘context-specific’ bioinformatics study. The chart is organized in the consecutive major steps labelled as 1 to 7, and it contains four columns; the first column shows the number of protein sequences before and the last column that of after the execution of each step (No seq INPUT and No seq OUTPUT), respectively. The second column shows the description of the steps, the third column the references to the steps, respectively. For details see ‘Materials and Methods.’ To carry out these steps, we have written a few Python-routines for the steps 1 through 3 and employed several open access programs (steps in light grey).

**Figure 2:**
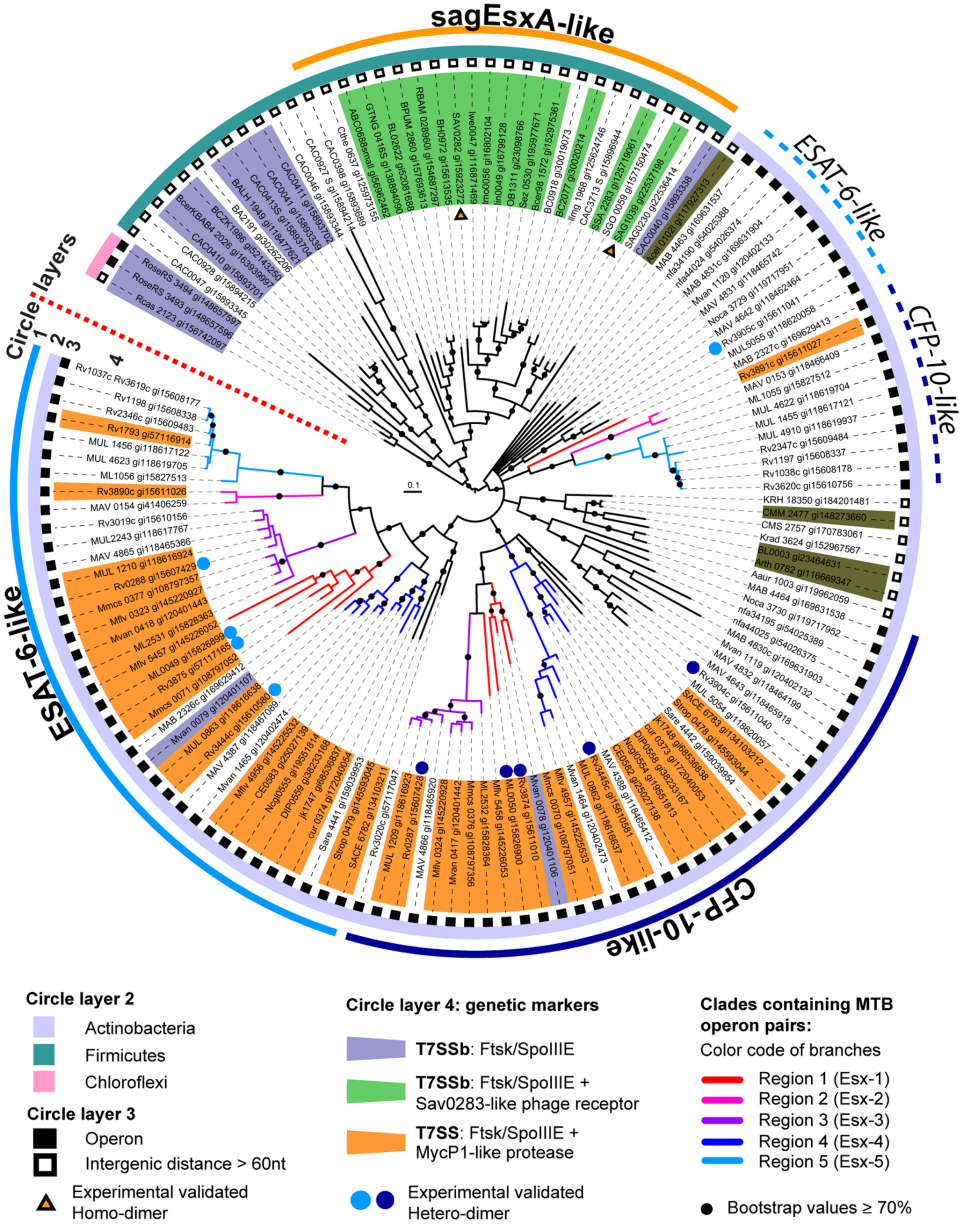
Estimated phylogenetic tree of the WXG-100 protein family consisting of three WXG100 subfamilies. The tree of WXG100 proteins was constructed in midpoint rooted presentation with three main clades: CFP-10-like (blue circular arc), ESAT-6-like (cyan circular arc) proteins and *sag*EsxA-like proteins (orange circular arc). The WXG100 gene pairs of *M. tuberculosis* occurring within the RD1-like gene clusters denoted as the regions (Esx) 1 to 5 are coloured accordingly along with the Rv-annotations (see subtitles). The annotations of the genes in close proximity to each of the WXG100 genes were manually analyzed and this information was also included to the tree. Two WXG100 genes with an intergenic distance of less than 80 nucleotides (according to the definition Roback et al. [47]) are considered to be encoded within a *bi*-cistronic operon (filled black squares on the circle layer 3), whilst mono-cistronic WXG100 genes are indicated by an unfilled squares. Those WXG-proteins whose oligomeric properties have been experimentally determined are marked with a triangle for homodimers and with pairs of blue dots for heterodimers. The second inner arcs show the phyla of the bacteria.

Proteins belonging to the WXG-100 family share less than 15% sequence identity with each other [6], which makes it extremely difficult to perform a meaningful alignment of protein sequences of this superfamily. To achieve a comprehensive sequence analysis and to link conserved residues to the structural data, we performed an iterative bio-informatics analysis. We combined all the available specific features known for this protein family and used a wide range of bio-informatics tools, with the results monitored in a step-wise manner (Fig. 1). It is worth noting that the collection of the available prokaryotic genome sequences, a priori, are somewhat biased, due to specific selection criteria such as bacteria habitats or cultivation properties and because the vast majority of bacteria have not yet been sequenced [17]. The first step in the sequence analysis was to collect a set of non-redundant WXG100 proteins. In the first step, an exhaustive search for WXG100 ORFs (Open Reading Frames) was performed using 940 fully sequenced prokaryotic genomes corresponding to ∼6 million ORFs of all phyla (Fig. 1). From this search we identified 2424 potential hits, which was reduced to 527 targets when the threshold for the predicted α-helical content was set to 40%. The genetic context of these targets was explored in the next step. When the occurrence of a *bi-*cistronic operon was taken into account and for those tandem genes containing the less stringent motif [W-H-L-F]-X-G, a further 153 putative WXG100 proteins were identified to give a total of 680 putative protein members for the WXG100 superfamily. All of the 22 known WXG100 proteins from *M. tuberculosis* (*tb*WXG100) were found to be among these 680 targets. To ensure that only truly homologous proteins were identified we used the classification tool CLANS [18]. The CLANS analysis resulted in a major cluster that contained a total of 183 proteins including all 22 WXG100 proteins from *M. tuberculosis* H37Rv (Fig. S2). We examined all the target proteins and could exclude a few following characterization by gene ontology (Figs. 1 and S2). An estimated phylogenetic tree was then calculated using the program MrBayes [19], including the 141 most diverse sequences out of 183 proteins (HHfilter was used for selection [20]). The resulting tree allowed us to understand the genetic relationship between the different WXG100 homologues (Fig. 2). As a result of this analysis we found that the targets originate almost exclusively from just two phyla, Actinobacteria and Firmicutes, with a limited number of targets originating from the phylum Chloroflexi (for further information see Materials & Methods and Fig. S2).

### Estimated Phylogenetic Tree Clusters the WXG100 Proteins into Three Subfamilies

The estimated phylogenetic tree is displayed as a circular representation and shows three distinct subfamilies of WXG100 proteins, the CFP-10-, ESAT-6- and *sag*EsxA-like proteins (Fig. 2, circle layer 1). The main outcomes of this iterative bioinformatics approach can be summarised as follows. Firstly, the CFP-10-like and ESAT-6-like protein clusters come exclusively from the phylum Actinobacteria and can be grouped into their own branches on the tree with relatively high bootstrap values (Fig. 2, black dots for ≥70%). A smaller number of WXG100 proteins from Actinobacteria with low bootstrap values cannot be grouped into the major ESAT-6- and CFP-10-like clades (Fig. 2, dotted arc on the outer most circle) but this does not affect the overall result regarding the organisation of this protein superfamily. Secondly, by examining every WXG100 gene in its genetic context we could clearly show that all WXG100 genes from the Actinobacteria phylum are encoded in a *bi-*cistronic operon containing a CFP-10-like gene followed by an ESAT-6-like gene without exception, whereas, all the *sag*EsxA-like genes occur as mono-cistronic genes in the bacterial genomes belonging to Firmicutes. To explore the relationship between WXG100 proteins and their secretion systems VII and VIIb, we looked for secretion system specific markers as previously described by Abdallah et al. [10] while exploring up to an additional eight ORFs up and downstream of the selected WXG100 genes (Fig. 2, denoted on the circle layer 4 and Fig. S1). This analysis showed that operons containing CFP-10 and ESAT-6-like genes are strictly associated with the T7SS, whereas the mono-cistronic *sag*EsxA-like genes are always clustered with the ancient version of the secretion system, T7SSb (Fig. 2 and Fig. S1).

### Sequence Alignments Reveal the Specific Features of the Three WXG100 Subfamilies

**Figure 3:**
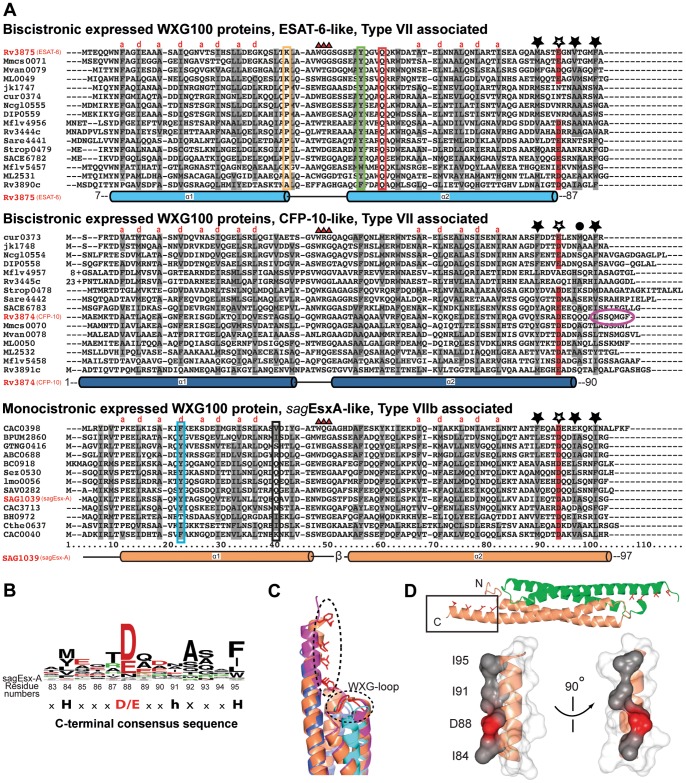
Alignments of the WXG100 subfamilies reveal conserved subfamily specific residues and generally conserved C-terminal residues pattern. (A) The position of helices, according to the structures of ESAT-6, CFP-10, and *sag*Esx-A, are shown below the alignments of each subfamily. The four-helix bundle requires mostly hydrophobic residues at the position of ‘a’ and ‘d’ of a helix turn consisting of the heptad helix repeat (a-b-c-d-e-f-g), shown as grey shading on the aligned residues. The key features of ‘ESAT-6-like’ subfamily (top panel): Shown are three highly conserved residues besides the almost invariant WXG motif (marked with red triangles), boxed in K/P38 (yellow), Y51 (green) and Q55 (red). Numbering of residues followed those of ESAT-6 (Rv3875). In the ‘CFP-10-like’ subfamily (middle panel), there are almost no conserved features, except for the C-terminal sequence conservation (marked with asterisks, filled with black for hydrophobic residues and unfilled for acidic residues), shared by all WXG100 superfamily members. In the ‘*sag*Esx-like’ subfamily (bottom panel), all residues involved in the inter-dimer interactions are hydrophobic except two residues, boxed in cyan and black. The gene IDs of the WXG targets are shown on each line. The numbers correspond to the locus of each genes depicted here. The bacterial species out of the phylum “Actinobacteria” are abbreviated as: Mmcs0071: *Mycobacterium sp*. MCS, Mvan: *M. vanbaalenii*, ML: *M. leprae*, jk: *Corynebacterium (C.) jeikeium*, cur: *C. urealyticum*, Ncgl: *C. glutamicum*, DIP: *C. diphtheria*, Mflv: *M.gilvum*, Sare: *Salinispora (Sa.) arenicola*, Strop: *Sa. tropica*, SACE: *Saccharopolyspora erythraea*, and those from the phylum “Firmicutes” as: CAC: *Clostridium acetobutylicum*, BPUM: *Bacillus pumilus*, GTNG: *Geobacillus thermodenitrificans*, ABC: *alkaliphilic Bacillus clausii,* BC: *Bacillus cereus*, Sez: *Streptococcus equi,* Lmo: *Listeria monocytogenes serovar*, SAV: *Staphylococcus aureus*, SAG: *Streptococcus agalactiae*, BH: *Bacillus halodurans*, Cthe: *Clostridium thermocellum*, respectively. (B) The C-terminal consensus sequence *HxxxD/ExxhxxxH* is shown as a sequence logo diagram. The residue at the eighth position is marked with ‘*h*’ indicating lower conservation on hydrophobic residues (see panel A). (C) Structural superposition of CFP-10 (blue), ESAT-6 (cyan), *sag*EsxA (orange) and *sauEsx*A (violet): Only the C-terminal helices along with the adjacent WXG loops facing towards helices are shown. For better visibility only the side chains of *sag*EsxA are shown. (D) The side chains of the conserved C-terminal residues decorate the same side of the C-terminal helix as observed in the structures of the WXG100 proteins, shown is that of *sag*EsxA (see text), marked with asterisks in panel A. To emphasize the structural feature, the C-terminal helix is shown in a surface representation, where the consensus hydrophobic residues are in grey and the acid residue is in red. The remaining residues (x) are shown in light grey.

**Figure 4:**
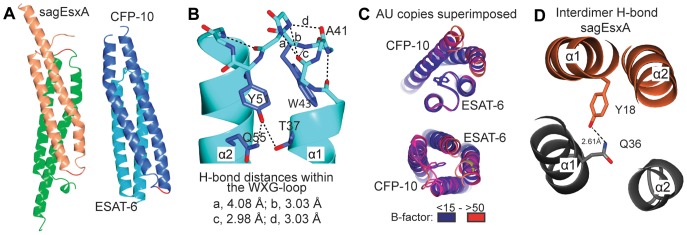
Structures of *sag*EsxA and CFP-10/ESAT-6 complexes, and comparisons of the loop conformation, as observed in the three WXG100 proteins. (A) The four-helix bundle structures of the homodimeric *sag*EsxA and heterodimeric CFP-10/ESAT-6 complexes are shown. (B) The WXG motif-containing loops of ESAT-6 showing an extended hydrogen-bonding network as indicated by dashed lines and labelled with their hydrogen bond donor-acceptor distances. (C) Comparisons of the loops of CFP-10 and ESAT-6. The asymmetric unit (AU) of CFP-10/ESAT-6 crystal contains two copies of the heterodimer. The view shows down towards the central long axis of the dimer. The relation of top to bottom panel views are 180° rotation around central short axis of the dimer, showing the WXG containing loop of ESAT-6 (top) and that of CFP-10 (bottom). Superimpositions of the structures of the AU content show that the WXG containing loops of ESAT-6 exhibit lower B-values and overlap better than that of CFP-10. (D) A hydrogen bond interaction formed by Y18 and Q38 at the inter-dimer interface of *sag*EsxA is shown.

Based on the results of the phylogenetic tree analysis, we carried out an alignment of sequences within the individual subfamilies. Only the subsets of the proteins with the most diverse sequences are displayed in Fig. 3A. To investigate how specific sequence motifs translate into specific structural features, we determined the atomic structures of two representative complexes, the heterodimeric CFP-10/ESAT-6 complex and the homodimeric *sag*EsxA assembly. Statistics of the crystallographic data and refinement are presented in Table 1. The overall structures of the two protein complexes are similar, with both forming a four-helix bundle, in which each monomer consists of a helix-loop-helix structural motif (Fig. 4A). An electrostatic surface potential representation shows that the complexes are relatively acidic (Fig. S5). However, an interesting result of the structure was that the highly conserved WXG motif of the loop region shows the least structural conservation (Fig. S3A). The specific characteristics of the subfamilies are described in detail below.

**Table 1:** Crystallographic statistics.

Complex	CFP-10/ESAT-6	*sag*EsxA	*sag*EsxA	*sag*EsxA
	(3FAV)	SeMet	native I (3GVM)	native II (3GWK)
Wavelength (Å)	1.033	0.979	1.000	0.900
Space group	C2	P2_1_2_1_2	P2_1_2_1_2	P6_5_22
*a, b, c* (Å)	160.34, 23.93, 83.86	132.44, 132.52, 43.49	133.10, 132.53, 43.67	76.58, 76.58, 149.74
β (°)	94.36			
Resolution range (Å)^a^	20-2.15	50.0-2.50	50-2.10	50-1.30
	(2.21-2.15)	(2.57-2.50)	(2.21-2.10)	(1.33-1.30)
Measured reflections	58053	138669	263051	678917
Unique reflections^a^	17336 (978)	50045 (3650)	43011 (3128)	62862 (4438)
*R*_sym_ b(%)^a^	5.5 (19.7)	4.0 (13.1)	9.1 (50.7)	5.3 (47)
*R*_meas_ c(%)^a^	6.5 (26.1)	4.9 (16.0)	10 (55.4)	5.5 (49.9)
Completeness	95.5 (73.4)	98 (94.7)	99.9 (99.9)	97.6 (95.2)
*<I/σ(I)>* g	14.4 (4.2)	19.8 (7.8)	16.8 (3.8)	23 (5.1)
Multiplicity^a^	3.3 (2.2)	2.8 (2.7)	6.1 (6.1)	10.8 (7.5)
Average B (Å^2^), overall/main chain	33.9/32.1		32.1/30.3	20.7/18.3
*R*_cryst_ d(%)	20.1		18.5	14.8
*R*_free_ e(%)	23.3		21.5	18.8
RMSD from ideal				
Bond length (Å)	0.023		0.008	0.017
Bond angles (°)	1.34		0.89	1.32
Dihedral angles (°)	17.8		19.0	17.4
Ramachandran plot^f^				
Most favoured region (%)	99.3		98.6	98.4
Additional allowed regions (%)	0.7		1.4	1.6

a Numbers given in brackets are from the last resolution shell.

b *R_sym_* = (Σ_hkl_Σ_i_|I_i_(hkl)-<I(hkl)>)/Σ_hkl_ΣI_i_(hkl), where I_i_(hkl) is the intensity of the *i*th measurement of reflection (hkl) and <I(hkl)> is the average intensity.

c *R_meas_* = (Σ_hkl_ (sqrt(N_hkl_/(N_hkl_-1))Σ_i_|I_i_(hkl)-<I(hkl)>)/Σ_hkl_ΣI_i_(hkl), where I_i_(hkl) is the intensity of the *i*th measurement of reflection (hkl) and <I(hkl)> is the average intensity.

d *R_work_* = (Σ_hkl_|Fo-Fc|/Σ_hkl_Fo where Fo and Fc are the observed and calculated structure factors.

e *R_free_* is calculated as for *R_work_* but from a randomly selected subset of the data (5%) which were excluded from the refinement [48].

f Ramachandran et al., 1963 [49].

g *I* is the integrated intensity and σ(*I*) is the estimated standard deviation of that intensity.

#### The ESAT-6-like subfamily

Sequence alignments of the ESAT-6-like proteins reveal distinctive features in contrast to the CFP-10-like and *sag*EsxA-like subfamilies. In ESAT-6-like proteins, Q55 is strictly conserved (Fig. 3A, red box) and its side chain interacts with the highly conserved Y51 (Fig. 4B and Fig. 3A, green box). The side chain of Y51 is located at the interface between the α1 and α2 helices of ESAT-6 where it forms an inter-helical hydrogen bond with the hydroxyl group of T37 and an intra-helical hydrogen bond with the side chain amide group of Q55 (Fig. 4B, and Fig. S5A). Furthermore, the loop residues (L39-S48) between helices α1 and α2 containing the WXG motif form extensive intra-molecular interactions. In particular, the indole ring of the conserved W43 is oriented towards two helices and together with Y51, W43 blocks the top interface of the helices α1 and α2. In addition, the WXG-motif of ESAT-6 forms an extended hydrogen-bonding network, where the main chain amine groups of W43-G44-G45 form hydrogen bonds to the main chain carbonyl groups of A40 and A41 (Fig. 4B). The remaining residues of the loop G45-S48 form a type I β-turn. Finally, residue 38 at the transition between the helix α1 and the loop is frequently a proline in many ESAT-6-like sequences (Fig. 3A, yellow box), although in ESAT-6 from *M. tuberculosis* it is a lysine (Fig. 3A). The three residues, Y51, Q55 and P/K38 show the highest degree of conservation after the conserved WXG motif, with the structural data suggesting that these three residues are the key determinants of the WXG loop conformation in ESAT-6. It is worth noting that such hydrogen bonding networks do not exist in the structures of CFP-10 or the homodimeric *sag*EsxA.

#### The subfamilies CFP-10 and *sag*EsxA

The CFP-10 subfamily is structurally more closely related to the *sag*EsxA than to the ESAT-6 subfamily. This is reflected by a root mean square deviation (r.m.s.d) of 2.07 Å for a superimposition of the core residues of CFP-10 (residues 11–83) and *sag*EsxA (residues 11–83), compared to an r.m.s.d. of 3.43 Å for the equivalent ESAT-6/*sag*EsxA superimposition (Fig. S3B). A significant difference between the CFP-10 and sagEsxA subfamilies and the ESAT-6 subfamily is to be found in the key loop between helices α1 and α2 which contains the essential WXG motif. In CFP-10, the WXG containing loop (residues 39–45) is three residues shorter than the corresponding loop of ESAT-6 (residues 39–48). In addition, it contains a 3_10_-helix with hydrogen bonds originating only from main chain atoms (Fig. S3A), whereas, the loop of *sag*EsxA (residues 43–48) consists only of a single tight β-turn. Therefore, both the CFP-10 and *sag*EsxA loops are considerably more flexible, which is reflected by the presence of different conformations in each of the copies of the asymmetric units. In contrast, the WXG loop of ESAT-6 adopts a more rigid conformation due to the extensive hydrogen bond contacts (Fig. 4B). This statement is supported by the significantly lower temperature factors (B-factors) for the ESAT-6 loop when compared with the equivalent CFP-10 loop (Fig. 4C). At the inter-dimeric interface, hydrophobic residues are prevalent in *sag*EsxA-like proteins although there is a pair of hydrophilic residues Y18 and Q36 within the hydrophobic core of *sag*EsxA (Figs. 3A, 4D and S5B). The side chains of these residues form hydrogen bonds within the hydrophobic core of the four-helix-bundle complex (Figs. 4D and S5B, inset) and the presence of these hydrogen bond pairs between Y18 and Q36 is indicative of the anti-parallel homodimer arrangement found in the *sag*EsxA subfamily of WXG100 proteins (Fig. 3A and 4D).

### Conserved C-terminal Sequence Pattern Forms a Specific α-helix Surface

The presented alignment of all WXG100 proteins revealed an additional conserved residue pattern at the C-termini that is present in all three sub-families (Fig. 3A). The consensus sequence of this pattern is ***H***xxx**D**/**E**xx***h***xxx***H*** *,* where ‘*H*’ stands for highly conserved hydrophobic and ‘h’ for less conserved hydrophobic residues, ‘x’ for any amino acid and “D/E” for either aspartic or glutamic acids, respectively (Fig. 3B). Interestingly, these residues are spaced about three residues apart, which correspond to a turn of an α-helix placing them all on the same face of the helix (Fig. 3A, marked with asterisks). In line with this hypothesis, the atomic structures of a homodimeric WXG100 protein (*sag*WXG100) from *S. agalactiae* and that of a heterodimeric protein CFP10/ESAT6 from *M. tuberculosis* revealed that these residues did indeed adopt an α-helical conformation. These signature residues decorate the same side of the C-terminal helix, which projects out of the core four-helix bundle while forming a distinctive surface (Fig. 3C and 3D).

### Oligomeric State of WXG-100 Proteins

Our finding that *sag*EsxA forms a homodimer led us to explore whether ESAT-6 is capable of forming a homodimeric complex as had been suggested in earlier reports [21]–[23]. Therefore, we expressed the CFP-10/ESAT-6 complex in *M. smegmatis* using the native operon including intergenic base pairs. The mono-cistronic *sag*EsxA was expressed in *E. coli* [13]. We first recorded CD (circular dichroism) spectra for the heterodimeric CFP-10/ESAT-6 complex and for the homodimeric *sag*EsxA to show that they were properly folded. The result in both cases was a CD spectrum typical for an α-helical protein with minima at 222 and 208 nm [24]. To study the thermal stability of the proteins, CD-spectra were recorded as function of temperature. Both samples exhibited a thermal unfolding transition temperature (Tm) between 40–50°C. The thermal unfolding was reversible, with the reversibility of the ellipticity in the case of *sag*EsxA (95%) being greater than that for the CFP-10/ESAT-6 complex (86%) which suggests that the *sag*EsxA is marginally more stable (Fig. S4). This result confirms the previous study of the reversible refolding property of the CFP-10/ESAT-6 complex [11], [24], and it shows that both types of dimeric complexes exhibit comparable thermal stability.

**Figure 5:**
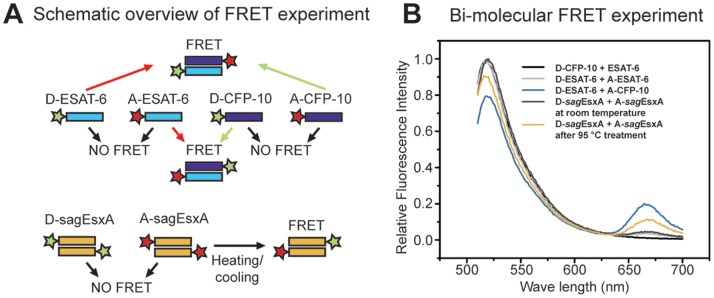
WXG100 proteins form dimeric complexes, studied using FRET. (A) Schematic diagram of the FRET experiments. Fluorescence donor, Alexa 488 (green), and fluorescence acceptor, Alexa 647 (red), are represented as stars. The Alexa fluorescence dye-conjugated proteins are indicated after their names along with the type of the Alexa dye, e.g. D-ESAT-6 instead of Alexa 488-ESAT-6. (B) The fluorescence spectra of the labelled proteins in those combinations, which were indicated in the schematic diagram A. Control contains only donor labelled protein (black). The donor/acceptor labelled ESAT-6 shows no FRET signal, also after heat de- and renaturation, indicating no homo-dimer formation (dark green). The donor labelled ESAT-6 and acceptor labelled CFP-10 gives a FRET signal, showing that CFP-10 and ESAT-6 spontaneously form a heterodimer (green). *sag*EsxA exhibits after initial mixing no FRET, but upon heat de- and renaturation there is reconstitution of FRET pairs (blue). For the FRET measurements the respective samples are mixed equimolar prior the measurements.

To investigate homo- versus heterodimer complex formation further, we carried out FRET (Förster Resonance Energy Transfer) measurements. The heterodimeric CFP-10/ESAT-6 complex (His_6_-CFP-10/ESAT-6) was decomposed into monomers under denaturing conditions and subsequently labelled chemically with amide active fluorescence dyes as described in Materials and Methods. The two monomers were conjugated separately with Alexa 488 (Donor-dye) or Alexa 647 (Acceptor-dye) to generate the four fluorescently modified monomers D-CFP-10, A-CFP-10, D-ESAT-6, and A-ESAT-6, respectively (Fig. 5A). There are theoretically four possible combinations of fluorescence samples as illustrated schematically (Fig. 5A). To detect the formation of dimers we recorded the static fluorescence intensity after mixing the donor labelled proteins with the acceptor labelled proteins. As a control we recorded a spectrum with a dimer where one monomer was labelled with the donor fluorophore and the second monomer was not labelled (Fig. 5B). A FRET signal could only be detected in those samples where donor-CFP-10 was mixed with acceptor-ESAT-6 or vice versa. These results demonstrate that CFP-10 and ESAT-6 exclusively form heterodimer and that heterodimer formation is a spontaneous process. The same experimental setup was employed to study the homodimeric protein *sag*EsxA. Interestingly, the *sag*EsxA FRET signal could only be detected after extensive heat treatment. This result shows that the homodimer is stable, and a FRET pair homodimer can only be reconstituted after heat dissociation (Fig. 5).

## Discussion

In recent years progress has been made concerning the nomenclature of the T7SSs and identifying a potential common secretion recognition sequence of the substrates for T7SS [5], [9], [25]. Despite these advances, the precise molecular function of the potent secreted T-cell antigens, CFP-10 and ESAT-6, and the protein secretion machinery for these prototypical WXG100 proteins remains poorly understood [25]. This prompted us to ask if we could gain some fresh insights by looking into the evolutionary origin of the WXG100 proteins. Due to the very low sequence conservation amongst the proteins of WXG100 protein superfamily, it has been difficult to obtain a robust alignment for these proteins [6]. Therefore, in the current work we used an iterative approach which combined the previously known features of CFP-10- and ESAT-6-like proteins with various available bioinformatics tools to generate an estimated phylogenetic tree for proteins of the WXG100 protein superfamily. This analysis was performed using the 940 completely sequenced prokaryotic genomes of all phyla that were available in the NCBI-databank as of November 2009. Despite screening the prokaryotic genomes of all phyla in the database, WXG100 protein superfamily members are found to originate mainly only out of two phyla, Actinobacteria and Firmicutes, in good agreement to the published data by Sutcliff [25]. A subsequently calculated estimated phylogenetic tree of WXG100 proteins is also in good agreement with the evolutionary phylogenetic tree of the mycobacterial phylum, Actinobacteria [26]. However, the novelty of this contribution is that by using the iterative bio-informatics approach, we can show that the WXG100 protein superfamily can be divided into three subfamilies: CFP-10-, ESAT-6- and *sag*EsxA-like. In addition, the phylogeny analysis of WXG proteins indicates that the Actinobacteria might be evolutionarily closer to the bacterial phylum Firmicutes than previously thought [27]. Subsequent analyses of the genetic organisation of WXG100 genes show that CFP-10- and ESAT-6-like genes are always encoded together on a *bi*-cistronic operon in contrast to the mono-cistronic s*ag*EsxA-like genes. This strongly suggests that CFP-10- and ESAT-6-like proteins are mutually dependent as they are encoded as tandem genes on an operon confirming the fact that these proteins are soluble only when they are in complex [24]. The phylogenetic tree suggests that CFP-10- and ESAT-6-like proteins are derived from a mono-cistronic ancestor (*sag*EsxA-like protein) and most probably co-evolved into unique sub-families after a gene-duplication event. The resulting sequence alignments reveal an interesting pattern of sequence conservation, which could be closely correlated with the atomic structures. This combined bioinformatics and structural analyses showed that the loop containing the highly conserved WXG motif exhibits the greatest structural diversity in the entire protein. Indeed, a different loop conformation is observed for each of the three WXG100 protein subfamilies (Fig. 4B and Fig. S3). Exactly why a different conformation is required for each subfamily remains unclear but we could show that the more rigid ESAT-6 loop is due to the presence of an extensive hydrogen bonding network which is largely absent in both the CFP-10 and *sag*EsxA subfamilies. This network of hydrogen bonds is probably the basis for the reported molten globular structure of ESAT-6 in solution in absence of the complex partner, CFP-10 [24]. This result fits quite well with the published NMR structure of the CFP-10/ESAT-6 complex from *M. tuberculosis*. However, our structural analysis shows that the nature of the molten globular structure of ESAT-6 protein is not based on the hydrophobic core [28] but rather on the hydrogen bonding network.

The *sag*EsxA-like proteins form a homodimer and the structure of the *sag*EsxA shows that the driving force for the interdimer interactions is principally hydrophobic in nature. However there are pair of mutations which are conserved among the proteins of the *sag*EsxA-like subfamily (Fig. 3A). Therefore, at some point in evolution an unfavourable mutation was rescued by a second mutation at a complementary point in the same molecule that could be accommodated in an anti-parallel homodimer. As a result, this conserved pair of mutations can be seen as a fingerprint of anti-parallel homodimeric WXG100 proteins. The new results presented here unambiguously show that the potent and widely studied T-cell antigens, CFP-10 and ESAT-6 can only form a heterodimeric complex. Previously, we have shown that other CFP-10- and ESAT-6-like heterodimers from *M. tuberculosis, M. smegmatis,* and *M. leprae* could be co-expressed and that they too form heterodimeric complexes [13]. Our results show that CFP-10-like and ESAT-6-like proteins group in the separated clades of the phylogenetic tree and that they are always encoded in an operon. This suggests that the determinant of the oligomeric state of the WXG-100 proteins is encrypted at the genetic level. That is to say, if two WXG-100 genes are encoded on a *bi*-cistronic operon they can only form a heterodimeric complex, whereas a gene product of a mono-cistronic WXG-100 gene forms a homodimeric complex. These results provide an explanation for why ESAT-6 cannot self-associate to form homodimers as previously suggested [21]–[23] and confirm the results of Renshaw *et al.* which showed that CFP-10 and ESAT-6 are only stable as a heterodimeric complex [24]. With regard to the single WXG-100 proteins, there have been a number of reports as to whether or not they can form heterodimers. For example, the two mono-cistronic genes, *sau*EsxA and *sau*EsxB from *S. aureus* have been suggested to form a heterodimer that is analogous to the ones from *M. tuberculosis* [8]. However, the determined crystal structure of *sau*EsxA from *S. aureus* revealed a homodimeric structure. This was despite the fact that the authors attempted to co-crystallize *sauEsx*A and *sauEsx*B together [12]. In this experiment the crystallization drop contained both proteins but only *sauEsx*A crystallized as a homodimer, similar to the structure of *sag*EsxA that we report here. This most likely means that the mono-cistronic gene products of *sauEsx*A and *sauEsx*B, both form homodimers. Based upon the results presented here we can accurately predict the oligomeric form of any WXG-100 protein simply by looking to see if it is encoded by a *bi*-cistronic operon or mono-cistronic gene.

In addition, it is tempting to propose an extended secretion signal for members of the T7SS and T7SSb, which has the structural properties of an α-helix. Champion *et al.* reported that the seven consecutive C-terminal residues of CFP-10 were needed for the secretion of the CFP-10/ESAT-6 complex (Fig. 3A, oval circle on CFP-10 sequence) [14], despite the majority of these seven residues are not conserved among WXG100 proteins. Recently, Daleke and co-workers reported a novel secretion signal YxxxD/E for T7SS, where, the three residues spacing between the crucial residues Y and D/E is of key importance [9]. The authors initially found the pattern on the PE proteins, e.g. PE25, but they also described the same pattern as being present on CFP-10-like proteins and other known T7SS substrate, such as EspB. Furthermore, Champion and co-workers analysed all 25 C-terminal residues of CFP-10 using alanine-scan mutation accompanied with the yeast two-hybrid approach to detect their importance in interaction with ESAT-6 and EccCb (Rv3871). Their result showed that the D87 of the residue pattern YxxxD (residues 83–87) is important for interaction with EccCb, but it does not perturb the dimer formation with ESAT-6 (Supporting figure 1 of Champion et al. [14]). In light of our findings and combining the reports by those two groups, we would like to propose: The structural basis for this observation is that the motif YxxxD/E forms part of a turn of an α-helix. Indeed, with the exception of EspA, all other reported T7SS substrates, such as EspB (81-**Y**GEV**D**EEAATAL-92), EspC (87-**Y**SEA**D**EAWRKAI-98), LipY (88-**Y**AAA**E**LANASLL-99), and PE25 (87-**Y**ATA**E**ADNIKTF-98) share the extended consensus motif, presented here. Furthermore, the alignment presented here extends the sequence pattern, YxxxD/E, for two additional turns of the α-helix possessing a consensus motif (*H*xxxD/Exx*hx*xx*H),* which is shared by all WXG100 proteins. As the side chains of these signature residues decorate the same side of an α-helix, we propose that this might be representing a common binding motif for protein targets secreted by the T7SS/b pathway. This feature is present in the structures of the CFP10/ESAT-complex structure (PDB: 3FAV), *sag*EsxA (PDB: 3GVM and 3GWK, this work and Fig. 3C/D) and *sauEsx*A (PDB: 2VS0 and 2VRZ [12]). Therefore, the endogenous receptor of the T7SS might recognize its substrates by a helical surface, which combines both a hydrophobic surface and an acidic residue patch (Fig. 3C/D). Together with the extended C-terminal secretion signal and the fact that the WXG-loop is located in close structural proximity to the secretion signal we can speculate that these two parts form an interaction surface to the other binding partner thereby fulfilling its function.

Very recently, Anderson and co-workers reported on the oligomeric states of four potential WXG100 proteins from *S. aureus*: *sau*EsxA, -B, -C, and -D, although only A and B possess the WXG-signature motif [29]. In addition, the authors reported that the proteins *sau*EsxB and *sau*EsxD form a heterodimer and that the last six residues of *sau*EsxD constitute the secretion signal. However, the results of a mutation study showed that the residues Y and E in the YyniE motif of the last six C-terminal residues of *sau*EsxD were not important for secretion. According to our results, the C-terminal residues of *sau*EsxD in question should be extended from the residues 85 onward: 85-***F***FEA***D***EH***W***GTE***F***AKLYYNIEG-105. These residues contain the consensus motif ***H*** *xxx* ***D/E*** *xx* ***h*** *xxx* ***H*** which precede the last six residues that form an additional YxxxD/E motif. As this second YxxxD/E motif (underlined) is outside of the consensus motif for WXG proteins, which we report in this contribution, this might afford an explanation for the contradictory results obtained by mutating the Y and E in the motif YyniE.

Daleke and co-workers proposed a dual signal for the targeting of T7SS-substrates. It could well be that all WXG-loops adopt identical structures upon binding to their interaction partners or alternatively that the different WXG-loop structures observed here determine the specificity for their ‘own’ T7SSs. The interacting partners might utilize the WXG-loop to distinguish between the different WXG-100 proteins, since most WXG100 genes are encoded within ‘own’ gene clusters assembling their ‘own’ secretion machineries. Hence, the different structures of the WXG-loops might be a clue as to how the five T7SSs of *M. tuberculosis* discriminate between their own substrate proteins to be secreted. Sundaramoorthy et al. [12] have proposed that the WXG100 proteins could be acting as an adaptor protein which functions as a “bridge” to the host cell surface. Ize & Palmer stated that the CFP-10/ESAT-6 proteins could themselves be part of a secretion machinery [30]. It should be noted that because WXG100 protein complexes exist either as anti-parallel homo- or heterodimers the proposed secretion signals will be located at both ends of any such complexes. This means that the protein complexes exhibit a two-fold symmetry with regard to the secretion signal. In the case of a heterodimer the signals are not completely symmetrical, although it is an extremely exciting concept to combine the transport of the substrates with a symmetrical molecular feature versus a vectorial transport over the cell membrane.

In summary, our work may offer a basis upon which to unravel the mechanism of substrate recognition which is the first step of the T7SS secretion cascade. The T7SS must be important for pathogenic organisms, since it occurs five fold in the genome of *M. tuberculosis*, devoting approximately one percent of its genetic material. The elucidation of this secretion mechanism will be one of the keys to understand the ‘host-pathogen’ interaction of tuberculosis infection, since *M. tuberculosis* secretes potent T-cell antigens via this secretion system.

## Materials and Methods

Experimental procedures including cloning, expression, purification and crystallization are described in the section ‘Materials and Methods S1’. Further biophysical methods, CD and FRET, are also described in this section.

### Data Collection, Structure Determination and Refinement

Data collection of the CFP-10/ESAT-6 complex was carried out at 100 K with flash-cooled crystals. Native data of the CFP-10/ESAT-6 complex crystals were collected on the BM14 beamline at the ESRF using a MAR225 CCD detector. The reflections were indexed and their intensities scaled using the XDS program package [31]. Molecular replacement (MR) was performed with the program Phaser [32] using a truncated version of the NMR structure as a search model (PDB:1WA8). The 28 NMR-models were truncated to the core of the four-helix bundle (50% of the model, CFP10: Q13-36, A46-R77, ESAT-6: A13-L36, A50-I76) and used as the MR-search model. The program Phenix.refine was used for the structure refinement applying TLS (Translation/Libration/Screw-motion) method for each molecule in the AU [33]. The tracing of the electron density map was carried out manually using the program Coot [34] in successive steps of refinement and building. The *sag*EsxA complex crystals were briefly soaked in 3 M of Na-malonate pH 7.5 and flash-cooled. The native data from *sag*EsxA complex crystals were collected at the SLS beamline X06DA, using a MAR225 CCD detector at a wavelength of 1.00 Å. Diffraction data of SeMet-derivatized crystals were collected at the ESRF beamline, ID29, equipped with a ADSC Quantum Q315r detector at a wavelength of 0.9792 Å (peak). The structure solution was carried out using the SAD protocol of Auto-Rickshaw an automated crystal structure determination platform [35]. Briefly, the platform carried out the following steps: The input diffraction data from XSCALE were automatically prepared and converted into SCALEPACK format for use in Auto-Rickshaw using programs of the CCP4 suite [36]. F_A_ values were calculated using the program SHELXC [37]. Based on an initial analysis of the data, the maximum resolution for substructure determination and initial phase calculation were set to 3.0 Å. Two selenium positions out of the maximum number of 4 heavy atoms were found using the program SHELXD [38]. The correct hand for the substructure was determined using the programs ABS [39] and SHELXE [37]. The occupancy of all substructure atoms was refined using the program MLPHARE [36]. The initial phases were improved using density modification and phase extension using the program RESOLVE [40]. A partial α-helical model was produced using the program ALBE, an ARP/wARP module for tracing helices and strands. Auto-Rickshaw produced the partially built model having 187 residues out of the total number of 384 residues for the four molecules. This model was subsequently resubmitted automatically to Auto-Rickshaw using the MRSAD protocol [41] combining SAD data with the partially built model to resolution 2.5 Å. The calculated electron density was of sufficient quality to let ARP/wARP [42] build 87% of the residues. The initial model from ARP/wARP was used as search model for MR using higher resolution native data in the P2_1_2_1_2 and P6_5_22 space groups by the program Phaser [32]. The final refinement and building was completed with Phenix.refine [33] and Coot [34]. For the high-resolution structure at 1.3 Å, anisotropic thermal parameters were refined.

### Iterative Bioinformatics Analysis (Fig. 1)

The context-specific bioinformatics analysis involved seven consecutive analysis steps, while employing several known molecular features (Steps 1–3), and for which we wrote Python routines (Python scripts can be provided on request). For subsequent steps we employed open access bioinformatics tools. A set of WXG100 protein sequences was collected from the 940 bacterial genomes available in the TiGR database as of November 2009 using following five steps. **Step 1**: Sequences of lengths between 85–135 containing a “W-X-G” motif +/−10 residues from the midpoint were harvested out of ca. 6 millions ORFs originating from 940 bacterial genomes of all phyla. **Step 2**: Proteins containing a predicted alpha helical content below 40% according to the program Predator output were discarded [43]. **Step 3**: All sequences containing a central positioned W-X-G-motif were selected, as well as all their neighbouring genes. A few tandem genes of WXG100 proteins contained [H/L/F]-X-G. **Step 4**: CLANS, a classification tool based on all against all BLAST similarities was used to investigate the WXG100 proteins of step 3 [18]. The scoring matrix BLOSUM65 was used with its default settings. The clustering was viewed in CLANS. The attraction values were iteratively set until all WXG100 proteins from *M. tuberculosis* were clustered together. The resulting attraction value was 0.0165. **Step 5**: To reduce redundancy, the sequences sharing more than 90% identity were removed (HHfilter [20]). The genetic environment +/−10 ORFs up and downstream of each selected gene product was explored for adjacent proteins with homologies to the Ftsk/SpoIIIE protein family, Rv3883c MycP1 protease and Sav0283-like phage receptors. The identification was based on clustering in CLANS using a BLOSUM80 scoring matrix having default settings and a clustering cutoff P-value of 10E-15. **Step 6**: **Calculation of an estimated phylogenetic tree,** The final collections of sequences were alignment with Clustal X (2.0.9) [44], where the gap extension penalty was set very narrow to ‘2’ in the multiple alignment parameters and other parameters were not changed. The resulting alignment was taken without any further adjustment. The optimal substitution model, WAG+I+G+F, were based on the software ProtTest [45]. The substitution model was used in the phylogenetic analysis carried out in MrBayes (v3.2) [19]. The Markov chain Monte Carlo analysis ran for 2×10^6^ generations, sampling every 100 generations, using four chains and a temperature parameter of 0.05. The data converged having a standard deviation of split frequencies less than 0.05 after 7×10^5^ generations. Only the last 75% of the calculated trees were kept to perform the Bayesian analysis. The final estimated tree was visualized in iTOL using the mid-point as root [46]. **Step 7**: **Alignment of WXG100 subfamilies**, Sequences connected to T7SS and T7SSb were selected for alignment. We subdivided the targets according to the resulting tree and carried out the alignments. The three subfamilies are: CFP-10-, ESAT-6- and *sag*EsxA-like protein subfamilies. Sequences from the estimated tree were used after applying a filter allowing less than 65% identity (HHfilter, [20]). The alignments were carried out as described above.

## Supporting Information

Figure S1 **Genomic organization of the gene clusters of type VII/VIIb secretion systems including associated WXG100 proteins.** Shown is a schematic representation of the gene products of the homologous RD1 region of *M. tuberculosis*. Top: Regions encoding the type VII secretion systems from selected *Actinobacterial* species: Depicted are all five regions of *M. tuberculosis* and each of the Esx-1 homologous regions from *M. leprae* and *M. smegmatis*. Bottom: Regions of type VIIb secretion systems from selected bacteria from the phylum *Firmicutes*. Both types of secretion systems contain a member of the FtsK/SpoIIIE family (violet) and at least one gene belonging to WXG100 superfamily (red boxed blue arrows). The direction of the transcription and the relative length of the gene products are indicated by coloured arrows. The figure is modified after Abdallah et al. [10].
(TIF)

Figure S2 **Clustering of WXG100 proteins using CLANS 2D-plot of the retrieved sequences.** The CFP-10- and ESAT-6-like pairs and *sag*EsxA-like are marked for reference. The clusters containing the genetic pairs of CFP-10- and ESAT-6-like proteins marked in the same coloured circles. The sequences in the main cluster presented in (A) are used for the phylogenetic tree analysis. (B) and (C) show the CLANS 2D plots when applied higher stringency criteria for the pair wise similarity, attraction values were increased to ≥0.03. There are several condensed clusters that do not take part in the WXG100 cluster. These are all false positives, primarily transcription factors, with no homology to WXG100 proteins and could be discarded following this analysis.
(TIF)

Figure S3 **Structure comparisons.** (A) Comparisons of the loop structures of CFP-10, ESAT-6 and *sag*EsxA, showing that the conformations of the loops and the position of the indole ring of W43 are diverse. (B) Overall pair wise superposition between the structures of ESAT-6, CFP-10, and sagEsxA, showing the ESAT-6 is very distinct from the other two proteins. Homologous C_α_ atoms (8–85 ESAT-6) from the four-helix-bundle core of the crystal structures were superimposed.
(TIF)

Figure S4 **CD-Studies of homo- and heterodimers.** Representative CD spectra and melting curves are shown. (A) The CD spectra of *sag*EsxA exhibit similar spectra to that of the CFP-10/ESAT-6 complex, showing that both proteins are highly α-helical. (B) The molar ellipticities are recorded as function of temperature. left panel, melting curves; right panel, renaturation curves.
(TIF)

Figure S5 **Electrostatic surface potential (ESP) representation of the complexes.** The complexes are shown in their calculated electrostatic surface potential (blue, positive; red, negative; white, neutral), middle panels. The complex is rotated 90° around the intermolecular axis indicated with black broken lines and one subunit is shown in ESP representation and the other traced as lines. The insets are showing the hydrogen bonds networks of CFP-10/ESAT-6 complex (top) and the pair of hydrophilic residues within the hydrophobic inter-dimer surfaces of *sag*EsxA complex, a signature pattern of this WXG100 subfamily. The figure was contoured using PyMOL with electrostatic potential contour settings: 0.5 V (blue) and −0.5 V (red).
(TIF)

Materials and Methods S1 **(a) Cloning, expression and purification, (b) Crystallization of CFP-10/ESAT-6 complex and** ***sag*** **EsxA, (c) Unfolding/refolding experiment, (d) FRET analysis, and (e) Structure data deposition.**
(DOCX)
