# Supplementary figures and images for: WXG100 Protein Superfamily Consists of Three Subfamilies and Exhibits an α-Helical C-Terminal Conserved Residue Pattern
Source: PLoS One. 2014 Feb 26;9(2):e89313. doi: 10.1371/journal.pone.0089313 (PMC3935865; doi:10.1371/journal.pone.0089313)

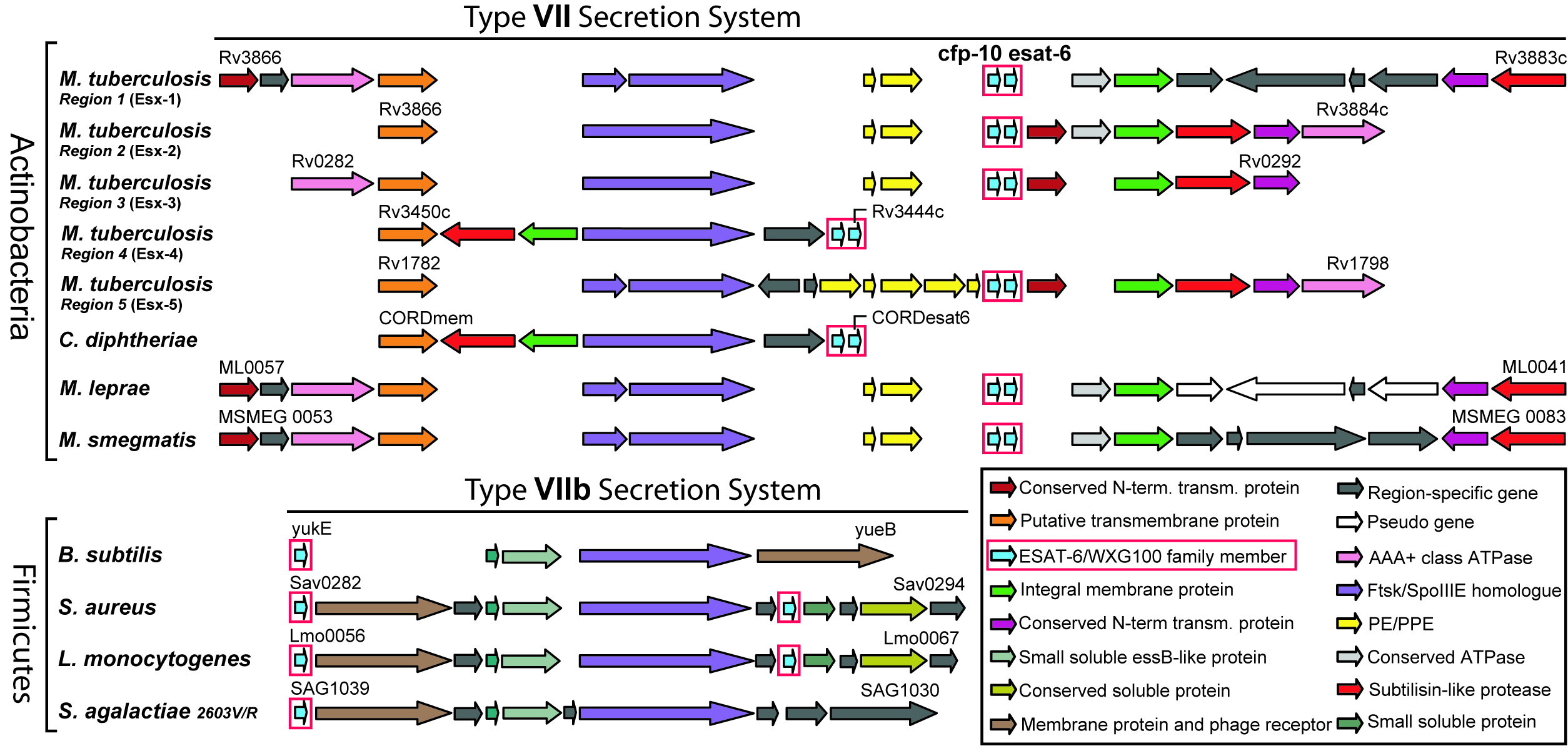

Supplement: Figure S1 — Genomic organization of the gene clusters of type VII/VIIb secretion systems including associated WXG100 proteins. Shown is a schematic representation of the gene products of the homologous RD1 region of M. tuberculosis. Top: Regions encoding the type VII secretion systems from selected Actinobacterial species: Depicted are all five regions of M. tuberculosis and each of the Esx-1 homologous regions from M. leprae and M. smegmatis. Bottom: Regions of type VIIb secretion systems from selected bacteria from the phylum Firmicutes. Both types of secretion systems contain a member of the FtsK/SpoIIIE family (violet) and at least one gene belonging to WXG100 superfamily (red boxed blue arrows). The direction of the transcription and the relative length of the gene products are indicated by coloured arrows. The figure is modified after Abdallah et al. [10]. (TIF) [file pone.0089313.s001.tif]

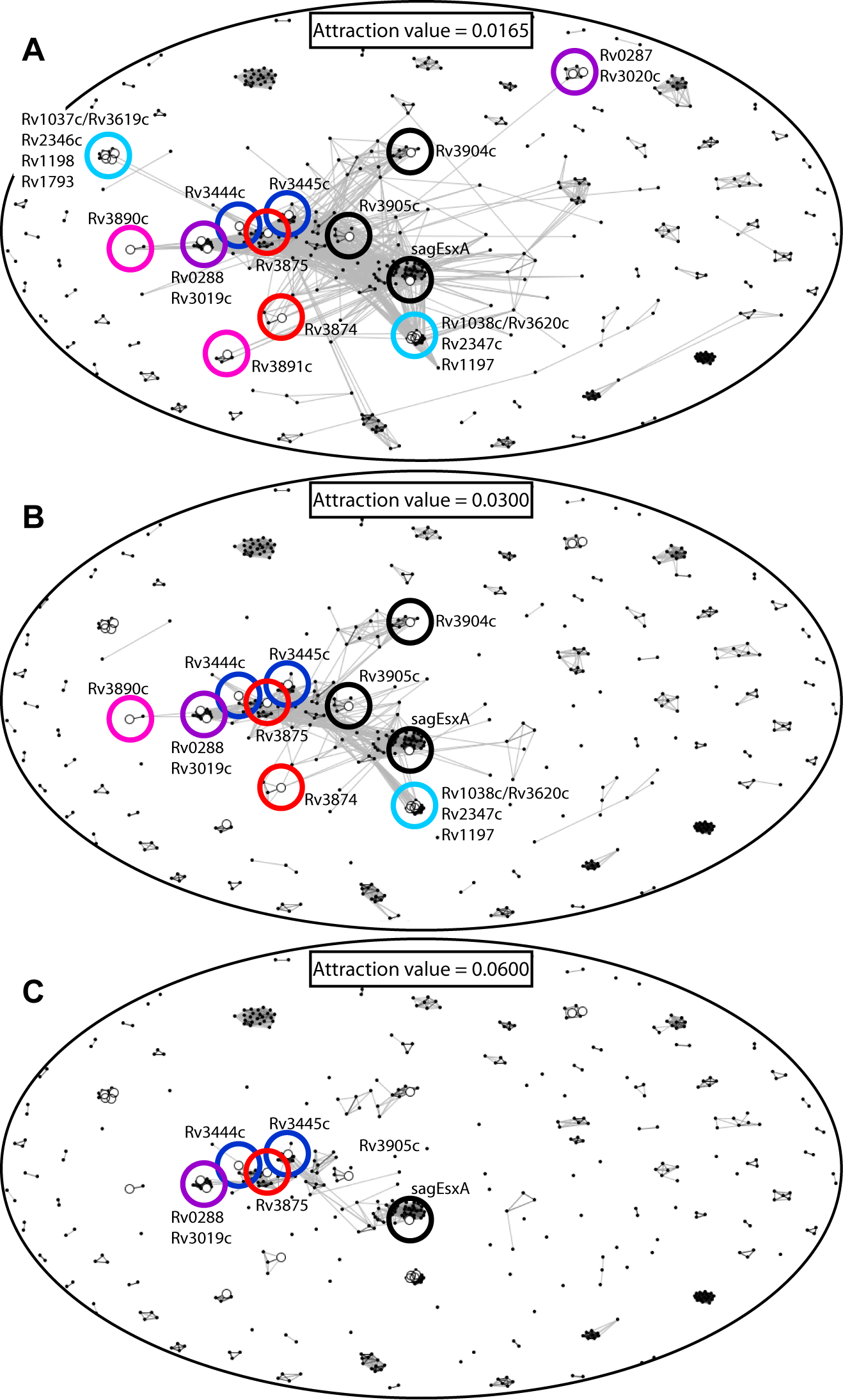

Supplement: Figure S2 — Clustering of WXG100 proteins using CLANS 2D-plot of the retrieved sequences. The CFP-10- and ESAT-6-like pairs and sagEsxA-like are marked for reference. The clusters containing the genetic pairs of CFP-10- and ESAT-6-like proteins marked in the same coloured circles. The sequences in the main cluster presented in (A) are used for the phylogenetic tree analysis. (B) and (C) show the CLANS 2D plots when applied higher stringency criteria for the pair wise similarity, attraction values were increased to ≥0.03. There are several condensed clusters that do not take part in the WXG100 cluster. These are all false positives, primarily transcription factors, with no homology to WXG100 proteins and could be discarded following this analysis. (TIF) [file pone.0089313.s002.tif]

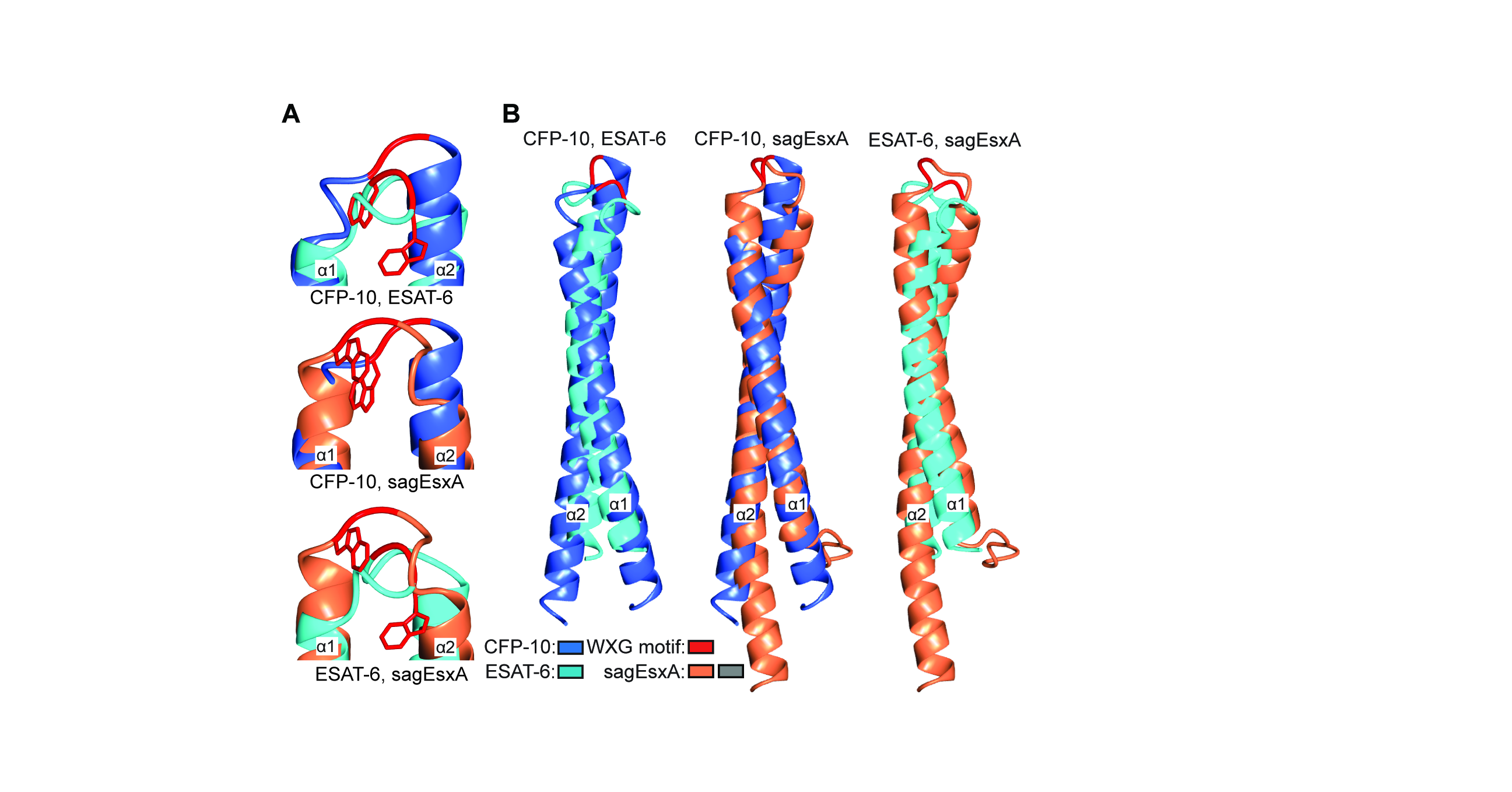

Supplement: Figure S3 — Structure comparisons. (A) Comparisons of the loop structures of CFP-10, ESAT-6 and sagEsxA, showing that the conformations of the loops and the position of the indole ring of W43 are diverse. (B) Overall pair wise superposition between the structures of ESAT-6, CFP-10, and sagEsxA, showing the ESAT-6 is very distinct from the other two proteins. Homologous Cα atoms (8–85 ESAT-6) from the four-helix-bundle core of the crystal structures were superimposed. (TIF) [file pone.0089313.s003.tif]

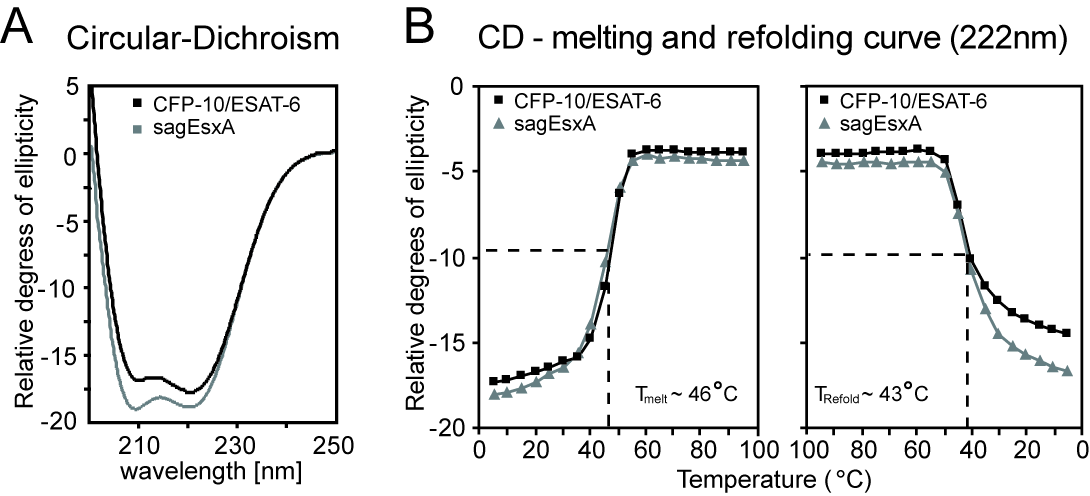

Supplement: Figure S4 — CD-Studies of homo- and heterodimers. Representative CD spectra and melting curves are shown. (A) The CD spectra of sagEsxA exhibit similar spectra to that of the CFP-10/ESAT-6 complex, showing that both proteins are highly α-helical. (B) The molar ellipticities are recorded as function of temperature. left panel, melting curves; right panel, renaturation curves. (TIF) [file pone.0089313.s004.tif]

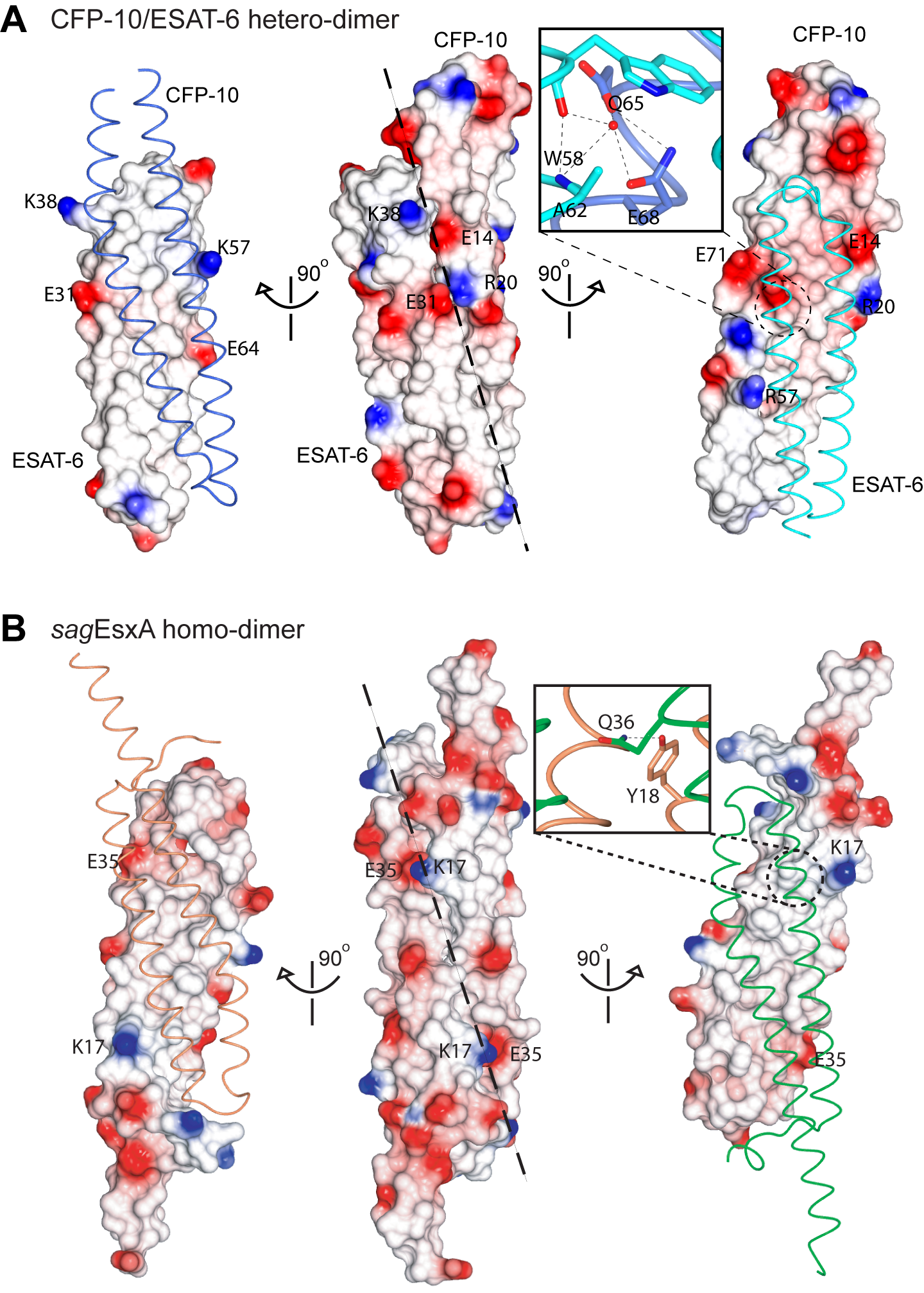

Supplement: Figure S5 — Electrostatic surface potential (ESP) representation of the complexes. The complexes are shown in their calculated electrostatic surface potential (blue, positive; red, negative; white, neutral), middle panels. The complex is rotated 90° around the intermolecular axis indicated with black broken lines and one subunit is shown in ESP representation and the other traced as lines. The insets are showing the hydrogen bonds networks of CFP-10/ESAT-6 complex (top) and the pair of hydrophilic residues within the hydrophobic inter-dimer surfaces of sagEsxA complex, a signature pattern of this WXG100 subfamily. The figure was contoured using PyMOL with electrostatic potential contour settings: 0.5 V (blue) and −0.5 V (red). (TIF) [file pone.0089313.s005.tif]
