# Supplementary material for: WXG100 Protein Superfamily Consists of Three Subfamilies and Exhibits an α-Helical C-Terminal Conserved Residue Pattern
Source: PLoS One. 2014 Feb 26;9(2):e89313. doi: 10.1371/journal.pone.0089313 (PMC3935865; doi:10.1371/journal.pone.0089313)
Supplement: Materials and Methods S1 — (a) Cloning, expression and purification, (b) Crystallization of CFP-10/ESAT-6 complex and sag EsxA, (c) Unfolding/refolding experiment, (d) FRET analysis, and (e) Structure data deposition. (DOCX) [file pone.0089313.s006.docx]

**Materials and Methods S1:**

**(a) Cloning, expression and purification**

The design of the CFP-10/ESAT-6 construct, its expression and purification was carried out as described previously [13]. In brief, the full-length operon containing the ORF of CFP-10 (Rv3874), ESAT-6 (Rv3875) and the intact intergenic base pairs of *M. tuberculosis* (*H37Rv*) were cloned into the pMyNT expression vector for expression in *M. smegmatis (Mc^2^155)*. The *sag*EsxA gene (SAG1039 of the *S. agalactiae* strain *2603V/R*) was synthesized by Geneart (Germany) and subcloned into the pETM11 vector. The expression of *sag*EsxA was carried out in the *E. coli* BL21(DE3)RIL strain. Proteins were purified using His_6_-tag affinity chromatography as follows. The extraction buffer was 50 mM Tris-HCl pH 8.0, 300 mM NaCl, 20 mM imidazole. The His_6_-tagged proteins were eluted from Ni-chelating column using the elution buffer containing 50 mM Tris-HCl pH 8.0, 150 mM NaCl and 500 mM imidazole. After the affinity purification the His_6_-tag was cleaved off using TEV protease (tobacco etch virus main protease) in extraction buffer supplemented with 10 mM DTT. Subsequently size-exclusion chromatography was performed using Superdex 75 (GE-Healthcare) to obtain a highly pure protein sample suitable for further study. The final buffer condition was 10 mM Tris-HCl, pH 8.0 and 100 mM NaCl. The SeMet (selenomethionine) derivatized *sag*EsxA protein was expressed in the methionine auxotrophic *E. coli* strain B834 (DE3), using M9 minimal media supplemented with 0.4% (w/v) glucose, 1 mM MgSO_4_, 0.3 mM CaCl_2_, 1 mg/L biotin, 1 mg/L thiamine and 50 mg/L of each amino acid exchanging methionine with SeMet. The subsequent purification was carried out following the same protocol as for the native protein.

**(b) Crystallization of CFP-10/ESAT-6 complex and *sag*EsxA**

The purified CFP-10/ESAT-6 homodimer and the *sag*EsxA homodimer were subjected to crystallized at a protein concentration >30 mg/ml using the EMBL-Hamburg high-throughput crystallization facility. Crystallization experiments were performed in 96-well plates using the sitting-drop vapour-diffusion method mixing equal volumes (300 nl) of protein solution and reservoir. The initial crystallization condition for the CFP-10/ESAT-6 complex contained 40% (v/v) PEG-600 (polyethylenglycol with molecular mass of 600), 100 mM imidazole, pH 8.0, 200 mM zinc acetate. The optimized crystallization condition was obtained by micro seeding and contained 33% (v/v) PEG-600, 100 mM imidazole, pH 6.5, 200 mM zinc acetate. The purified *sag*EsxA complex was concentrated to 10 mg/ml and was crystallized in a reservoir containing 100 mM Tris-HCl pH 8.0, 1.9 M (NH_4_)_2_SO_4_. SeMet-labelled *sag*EsxA protein was crystallized under the same conditions as the native protein.

**(c) Unfolding/refolding experiment**

The CD (circular dichroism) spectra of the CFP-10/ESAT-6 complex and the *sag*EsxA complexes were recorded in PBS-buffer (phosphate buffer saline) at a protein concentration of 4 µM. The melting/unfolding of the complexes were investigated by measuring the relative ellipticity at 222 nm over a temperatures range from 5-95ºC in steps of 5ºC. The refolding experiments were carried out by lowering the temperature from 95°C to 5ºC in steps of 5ºC. All experiments were carried out at least in triple repetition and performed using a Jasco J-810 spectropolarimeter.

**(d) FRET analysis**

To study the complex formation by using FRET, the His_6_-CFP-10/ESAT-6 complexes were first disrupted in 8 M urea and subsequently separated over a Ni-NTA column. ESAT-6 was collected in flow-through fraction and CFP-10 was eluted in 20 mM Tris‑HCl pH 8.0, 100 mM NaCl and 200 mM imidazole. The His_6_-tag of CFP-10 was removed as described in the section ‘Cloning, expression, purification and crystallization’. CFP-10, ESAT-6 and *sag*EsxA were extensively dialysed against the labelling buffer consisting of 100 mM Na-bicarbonate, pH 8.0, 100 mM NaCl, prior to the chemical coupling reaction with the fluorophores. For labelling a three-fold molar excess of Alexa-488-TFP or Alexa-647-TFP in dimethylformamide (DMF) was added and incubated overnight at 4 ºC. The N-terminal Alexa-488 (Donor=D=488) and Alexa-647 (Acceptor=A=647) labelled proteins were purified from free dye using a desalting column. The labelling efficiencies were determined prior to FRET measurements and were in the range 40 - 80 %. Each fraction was analysed by recording an absorption spectrum between 250 nm and 650 nm. The fractions with labelled proteins were pooled. For all FRET studies the final concentration of each donor-acceptor pair was 1 µM. The donor-acceptor pairs were mixed and measured at 20 °C. For reconstitution of the FRET-pair of *sag*EsxA, the fluorescent labelled proteins were first heated to 95 ºC for 4 min and allowed to cool to room temperature prior to the FRET measurement. For CFP-10 and ESAT-6 all potential FRET pairs were measured: D‑CFP‑10/A-CFP-10, D-CFP‑10/A‑ESAT‑6, A-CFP-10/D-ESAT-6 and D‑ESAT‑6/ A‑ESAT‑6. The emission fluorescence intensities were measured at the spectral range of 525 - 700 nm using Fluorolog3 (Horiba Jovin Yvon) with the excitation wavelength, λ_ex_ = 495nm.

**(e) Structure data deposition:** The atomic coordinates and structure factors have been deposited in the Protein Data Bank, www.pdb.org [PDB ID codes 3FAV (CFP-10/ESAT-6 complex), 3GVM (*sag*Esx‑A with resolution of 2.15 Å), and 3GWK (*sag*Esx‑A with resolution of 1.30 Å)
